# Erythrophages do not develop when lumbar CSF and blood samples are mixed in vitro

**DOI:** 10.1186/s12987-018-0116-3

**Published:** 2018-11-05

**Authors:** R. Dersch, D. Benkler, T. Robinson, A. Baumgartner, S. Rauer, O. Stich

**Affiliations:** 1 Department of Neurology, Medical Center-University of Freiburg, Breisacher Str. 64, 79106 Freiburg, Germany; 2 Department for Psychiatry and Psychotherapy, Medical Center-University of Freiburg, Freiburg, Germany; 3 MVZ Neurologie, Constance, Constance, Germany

**Keywords:** Lumbar puncture, CSF cytology, Subarachnoid hemorrhage, Traumatic tap, Erythrophages

## Abstract

**Background:**

Cerebrospinal fluid (CSF) analysis is a crucial method in the diagnostic process for suspected subarachnoid hemorrhage (SAH), especially when cerebral imaging is negative or inconclusive. CSF cytology (detection of erythrophages or siderophages) is used to determine whether a bloodstained CSF resembles a genuine SAH. Whether erythrophages may develop in vitro after a traumatic puncture in case of delayed CSF analysis is unclear. An in vitro development of erythrophages after traumatic puncture would diminish the diagnostic properties of CSF analysis. We assessed whether erythrophagocytosis is detectable in CSF after an imitated traumatic lumbar puncture.

**Methods:**

We mimicked a traumatic lumbar puncture by mixing surplus CSF with whole blood from the same patient. From this mixture, cytological specimens were obtained immediately and repeatedly at time intervals of 1 h, until 7 h after mixing, or until the mixture was exhausted. Each cytological specimen was microscopically examined independently by four experienced CSF cytologists for the presence of erythrophages.

**Results:**

We studied 401 CSF cytological specimens of 96 punctures in 90 patients. We could not identify any erythrophages in all cytological specimens. Fleiss’ Kappa for interrater-reliability was 1.0.

**Conclusions:**

We did not find evidence for an in vitro erythrophagocytosis after a mimicked traumatic lumbar puncture. Therefore, the occurrence of erythrophages in CSF cytology can be regarded as a reliable sign of an autochthonous bleeding in the subarachnoid space. Our results support the crucial role of CSF analysis in clinical practice in case of a suspected SAH but negative cerebral imaging.

**Electronic supplementary material:**

The online version of this article (10.1186/s12987-018-0116-3) contains supplementary material, which is available to authorized users.

## Background

Cerebrospinal fluid analysis is a crucial method in the diagnostic process if a subarachnoid hemorrhage (SAH) is clinically suspected but findings from cerebral imaging are negative or inconclusive. This is especially important in cases with an increased time interval between the suspicious clinical event and the cerebral imaging as the sensitivity of cerebral imaging declines significantly over time [1, 2]. When faced with prolonged time intervals between a suspicious event with acute severe headache and clinical examination, CSF analysis may be the only diagnostic tool to verify a SAH [3]. However, uncertainties can arise when an iatrogenic traumatic lumbar puncture is suspected, where trauma from the puncture needle can cause bleeding into the subarachnoid space. This artificial staining can be discriminated from a genuine intrathecal hemorrhage by clinical and laboratory tests. CSF findings supporting the diagnosis of a genuine subarachnoid hemorrhage are a positive “three-tube test”, xanthochromia of CSF supernatant, and the detection of indicative cell types for an intracerebral hemorrhage, such as erythrophages and siderophages in CSF cytology [4–6]. Several days after a subarachnoid hemorrhage, hematoidin crystals may be detected in CSF microscopy [6]. Cytological evidence of erythrophages or siderophages is highly specific and is regarded as evidence for a SAH [7].

Four hours after a subarachnoid hemorrhage, monocytes begin phagocytosis with ingestion of other cell types, mainly erythrocytes, and develop into erythrophages. Erythrophages are detectable approximately 4 h after a subarachnoid hemorrhage in CSF cytology. Erythrophages develop into siderophages by degrading ingested erythrocytes containing hemoglobin within the next 3–4 days [7, 8].

Whether erythrophages may develop in vitro after a traumatic lumbar puncture (LP) in case of a delayed CSF analysis is a matter of controversy in clinical practice. CSF should be analyzed quickly after LP. The quality of a cytological specimen decreases over time, as cells disintegrate in vitro over a period of several hours after the puncture. It is unsure whether surviving monocytes in CSF tubes could start to ingest erythrocytes and develop into erythrophages. This process would then lead to a false positive diagnosis of a subarachnoid hemorrhage after cytological CSF analysis. Evidence of in vitro development of erythrophages after traumatic puncture would therefore significantly diminish the diagnostic properties of CSF analysis in the setting of a suspected subarachnoid hemorrhage. We assessed whether an in vitro erythrophagocytosis after a mimicked traumatic LP is detectable in a CSF cytological specimen.

## Materials and methods

We studied CSF specimens from patients who underwent an LP as a part of a routine clinical workup for several neurological diseases or had repeated therapeutic LPs for diseases like benign intracranial hypertension. All patients gave their informed consent to LP and for the use of surplus CSF for this project. The study protocol was approved by the ethics committee of the Medical Center, University of Freiburg (EK-Fr 271/13). All data generated or analysed during this study are included in this published article. Clinical and demographic data (age, sex, diagnosis) was obtained from the medical records for each patient, as well as CSF routine parameters (white blood cell count (WBC), total protein, and, if available, albumin quotient and intrathecal immunoglobulin synthesis). Patients were excluded if they had a history of a subarachnoid or intracerebral hemorrhage. If the routine CSF cytology showed erythrophages in the initial routine analysis, the respective patient was excluded from further analysis.

Surplus CSF not needed for routine clinical analysis was harvested for immediate further handling at room temperature. To imitate a traumatic LP, surplus CSF was mixed with whole blood from the same patient collected at the same time point. Whole blood was mixed to CSF in a proportion of 2 μL whole blood/1000 μL CSF. If the LP happened to be traumatic by chance no additional whole blood was added to the CSF.

From this mixture, a cytological specimen was obtained immediately after mixing whole blood and CSF; additional single samples of cytological specimens were obtained at time intervals of 1 h until 7 h after mixing had passed or until the mixture was exhausted. During the incubation period the mixture was stored at room temperature. Each cytological specimen was performed using 1.2 mL of the mixture. Cytological specimens were stained using May–Grunwald staining.

Each cytological specimen derived from the mixture was microscopically examined by four experienced CSF cytologists (OS, AB, TH, and RD) independently, for the presence of erythrophages. If one cytologist detected erythrophages in a cytological specimen, the corresponding specimen was reviewed by all cytologists and the assessment was discussed accordingly. Concordance of the assessment was calculated using Fleiss’ Kappa.

## Results

Initially, CSF specimens from 96 patients were screened. Six patients had to be excluded due to various reasons. One patient withdrew consent after the LP was performed. One patient showed erythrophages in the initial routine CSF cytology and was therefore excluded from further analysis. Due to artificial changes, cytological specimens from four other patients could not be analyzed, as no leukocytes were detectable in the CSF sample. These patients were excluded from further analysis. In total, 401 CSF cytological specimens of 96 LPs in 90 patients were studied. The mean number of specimens per patient was 3.8 (SD 1.9). Demographic characteristics and results of routine CSF analysis of the included patients regarding their medical conditions are shown in Additional file 1: Table S1.

We obtained 50 cytological specimens at baseline directly after the puncture, 69 one hour after puncture, 88 two hours after puncture, 93 three hours after puncture, 65 four hours after puncture, 26 five hours after puncture, 9 six hours after puncture, and 1 seven hours after puncture. Of the included patients, 28 were suffering from neuroinflammatory diseases and 17 CSF samples showed inflammatory changes in terms of a pleocytosis in the initial routine analysis. In the cytological assessment, we did not identify erythrophages in any specimen. Fleiss’ Kappa for interrater-reliability was 1.0.

## Discussion

Whether an in vitro erythrophagocytosis exists in bloodstained CSF is a matter of controversy. An in vitro development of erythrophages after traumatic puncture would drastically reduce the diagnostic value of CSF analysis in clinical practice if a subarachnoid hemorrhage is suspected. Our study did not find any signs of erythrophagocytosis in CSF cytology after a mimicked traumatic LP in 401 CSF cytological specimens of 96 LPs from 90 patients. Fleiss’ Kappa for interrater-reliability was 1.0.

Whether in vitro erythrophagocytosis exists after traumatic LP has been scarcely investigated. The only available study used a comparable paradigm to our study to investigate in vitro erythrophagocytosis was in 1973 [9]. In this study performed by Oehmichen and Schütze, several different mix ratios of whole blood and CSF were applied in a total of 367 cytological specimens. However, the time interval between lumbar puncture and mixing of CSF with blood was not standardised and furthermore, cytological specimens were established using a sedimentation technique, which is no longer used currently. In contrast to our results, Oehmichen and Schütze found erythrophages in 17.5% of their cytological specimens of artificially bloodstained CSF. Cytological specimens were also obtained at delayed time points after mixing CSF and whole blood, but the authors state that this had no effect on the presence or amount of detectable erythrophages.

Methodological differences, like the use of the now-outdated sedimentation technique by Oehmichen and Schütze, may account for these differences. By using the sedimentation technique, the quantity of large cells like monocytes in CSF cytology is higher than in specimens obtained with a modern centrifuge. This may lead to more doubtfully altered cells which might be misinterpreted as erythrophages that are absent in cytological specimens obtained with current techniques.

Why erythrophages develop in SAH and not in CSF after an artificial traumatic lumbar puncture remains speculative. In SAH monocytes could be specifically activated by damaged choroid plexus epithelium or ependymal cells. Another explanation could be differences in temperature in vivo as compared to storing CSF samples at room temperature after lumbar puncture. Another issue could be longer incubation time of monocytes in CSF with contact to erythrocytes in vivo compared to the limited time after lumbar puncture until monocytes start to disintegrate in vitro.

## Conclusions

We did not find any erythrophages in CSF cytology after a mimicked traumatic LP. Therefore, the occurrence of this cell type in CSF cytology should be regarded as a reliable sign of an autochthonous bleeding in the subarachnoid space. Thus, our results support the crucial role of CSF analysis in clinical practice in case of a suspected subarachnoid hemorrhage and negative cerebral imaging.

## Additional file

**Additional file 1: Table S1.** Pathological conditions, demographic data and results from routine CSF analysis from included patients. Data is shown as mean with standard deviation.

## Abbreviations

CSF: cerebrospinal fluid
LP: lumbar puncture
SD: standard deviation
SDH: subarachnoid hemorrhage
WBC: white blood cell count
