# Supplementary material for: Erythrophages do not develop when lumbar CSF and blood samples are mixed in vitro
Source: Fluids Barriers CNS. 2018 Nov 5;15:31. doi: 10.1186/s12987-018-0116-3 (PMC6217771; doi:10.1186/s12987-018-0116-3)
Supplement: Supplementary file 1 — Additional file 1: Table S1. Pathological conditions, demographic data and results from routine CSF analysis from included patients. Data is shown as mean with standard deviation. [file 12987_2018_116_MOESM1_ESM.docx]

|  | n= | Sex (female %) | Age [years] | WBC [/µL] | CSF protein [mg/L] | Albumin quotient | Lactate [mmol/mL] |
| --- | --- | --- | --- | --- | --- | --- | --- |
| Multiple sclerosis/ neuroimmunological diseases | 18 | 14 (78%) | 44.2 (11.7) | 5.9 (5.6) | 457 (188) | 5.2 (2) | 1.4 (0.1) |
| Benign intracranial hypertension | 11 | 7 (63%) | 38.5 (10.9) | 1.8 (1.3) | 330 (114) | 4.1 (0.7) | 1.4 (0.1) |
| Normal pressure hydrocephalus | 19 | 9 (45%) | 77 (10.4 | 1.3 (1.1) | 589 (238) | 7.9 (3.2) | 2.4 (0.6) |
| Headache/migraine | 6 | 4 (66%) | 43 (9.4) | 1.5 (0.8) | 375 (131) | 4.8 (1.5) | n.a. |
| Seizure | 2 | 1 (50%) | 49.5 (10.5) | 1.5 (0.5) | 598 (30) | 7 (0.3) | n.a. |
| Meningitis/encephalitis | 8 | 4 (50%) | 62 (21) | 39.5 (44.2) | 691 (493) | 8.5 (4.8) | 1.7 (0.4) |
| Malignancies | 2 | 0 (0%) | 65.5 (1.5) | 521 (519) | 788 (332) | 9.5 (2.2) | 2.4 (0) |
| Motor neuron disease | 1 | 0 (0%) | 54 (0) | 1 (0) | 492 (0) | 6.2 (0) | n.a. |
| Polyneuropathy/ polyradiculopathy | 2 | 0 (0%) | 81 (16) | 1.5 (0.5) | 721 (79) | 8.5 (2) | 1.6 (0) |
| Ischemic stroke | 2 | 0 (0%) | 55.5 (7.5) | 1 (0) | 940 (341) | 13 (5.2) | 1.6 (0) |
| Other | 18 | 8 (44%) | 51 (13) | 2.3 (1.4) | 475 (208) | 6 (2.8) | 2.3 (1.2) |
| Table S1: Pathological conditions, demographic data and results from routine CSF analysis from included patients. Data is shown as mean with standard deviation in brackets. WBC: white blood cell count | | | | | | | |
